# A proposal of a new evaluation framework towards implementation of genetic tests

**DOI:** 10.1371/journal.pone.0219755

**Published:** 2019-08-05

**Authors:** Erica Pitini, Elvira D’Andrea, Corrado De Vito, Annalisa Rosso, Brigid Unim, Carolina Marzuillo, Antonio Federici, Emilio Di Maria, Paolo Villari

**Affiliations:** 1 Department of Public Health and Infectious Diseases, Sapienza University of Rome, Rome, Italy; 2 Division of Pharmacoepidemiology and Pharmacoeconomics, Department of Medicine, Brigham and Women's Hospital and Harvard Medical School, Boston, Massachusetts, United Sates of America; 3 Ministry of Health, Rome, Italy; 4 Department of Health Sciences, University of Genova, Division of Medical Genetics, Galliera Hospital, Genova, Italy; University of Toronto, CANADA

## Abstract

**Background:**

The existing frameworks for the evaluation of genetic and genomic applications clearly address the technical and clinical value of a test, but are less concerned with the way genetic services are delivered and organized. We therefore aimed to develop a comprehensive new framework that includes an assessment of service delivery.

**Methods:**

A new framework was built on the evaluation dimensions identified through a systematic review of the existing frameworks and a Delphi survey of Italian experts in public health genomics.

**Results:**

Our framework has four sections. The first two sections, respectively, guide the evidence collection process for the genetic test (analytic validity; clinical validity; clinical utility; personal utility) and its delivery models (organizational aspects; economic evaluation; ethical, legal and social implications; patient perspective). The third section guides the formulation of the research priorities to be addressed in future research. Finally, the fourth section suggests three criteria to summarize the collected evidence (net benefit, cost-effectiveness, feasibility).

**Conclusion:**

We have successfully developed an evaluation framework for the evaluation of genetic tests that includes an assessment of service delivery. It also introduces some neglected evaluation dimensions such as personal utility and patient perspective.

## Introduction

The expanding knowledge of the human genome and the consequently growing availability of genetic and genomic applications in recent decades are expected to revolutionize medicine [1]. The most significant promise lies in precision medicine, where medical decisions are tailored to an individual’s characteristics, including the patient’s genetic profile [2]. However, there is still a wide gap between reality and expectations, partly due to the absence of a completely satisfactory framework for distinguishing useful innovations from unwarranted interventions [3].

The existing frameworks for the evaluation of genetic and genomic applications rely mainly on the technical and clinical assessments described in the well-known ACCE (Analytic validity, Clinical validity, Clinical utility, Ethical, legal and social implications) model [3, 4]. Nevertheless, such frameworks lack a structured evaluation of the implementation issues and how these relate to the context in which the test will be used; i.e. how genetic services should be (re-)organized to implement and deliver the new test [3]. Now more than ever the precision medicine model is under the spotlight, and it appears clear that an evaluation limited to technical performance and clinical outcomes is no longer sufficient for effective “research translation”[5, 6]. This is particularly true for publicly funded healthcare systems, where equity and resource constraints are a major concern.

In Italy, the approval of genetic and genomics applications for funding by the National Healthcare System is largely unregulated. For this reason, the National Plan for Public Health Genomics has proposed, as a key objective, the development of a more comprehensive evaluation framework for genetic and genomic applications, which could support decision-makers in coverage decisions [7]. Our work was conducted as part of a project financed by the Italian Ministry of Health to implement this plan and aims to develop a comprehensive evaluation framework that includes an assessment of service delivery.

## Materials and methods

A new framework was developed by combining a systematic review and a Delphi survey. The systematic review was performed to review the need for a new framework and to provide a basis for its creation. It aimed to identify and compare the existing frameworks, with a particular focus on their evaluation dimensions (i.e. analytic and clinical validity, clinical utility, etc.). The Delphi survey was designed to support the systematic review and aimed to identify additional frameworks and to further explore the importance of their dimensions.

Throughout the paper, a genetic/genomic test is defined as an analysis of human chromosomes, DNA, RNA, genes, and/or gene products (e.g. enzymes or other proteins) that is primarily used to detect heritable or somatic mutations, genotypes, or phenotypes related to disease and health [8].

### Systematic review

The systematic review is described elsewhere [3]. In brief, we included any document that described an original evaluation framework for genetic tests. We searched the bibliographic databases PUBMED, SCOPUS, ISI Web of Knowledge, Google Scholar and Google for all English language articles between January 1990 and April 2017. This search was supplemented by exploring the websites of the main government agencies and research organizations involved in the evaluation of genetic tests. We screened the retrieved records by title, abstract and full text. From the included records, we extracted information about the evaluation frameworks, their evaluation dimensions and other methodological aspects.

### Delphi survey

#### Expert panel

**Fig 1:**
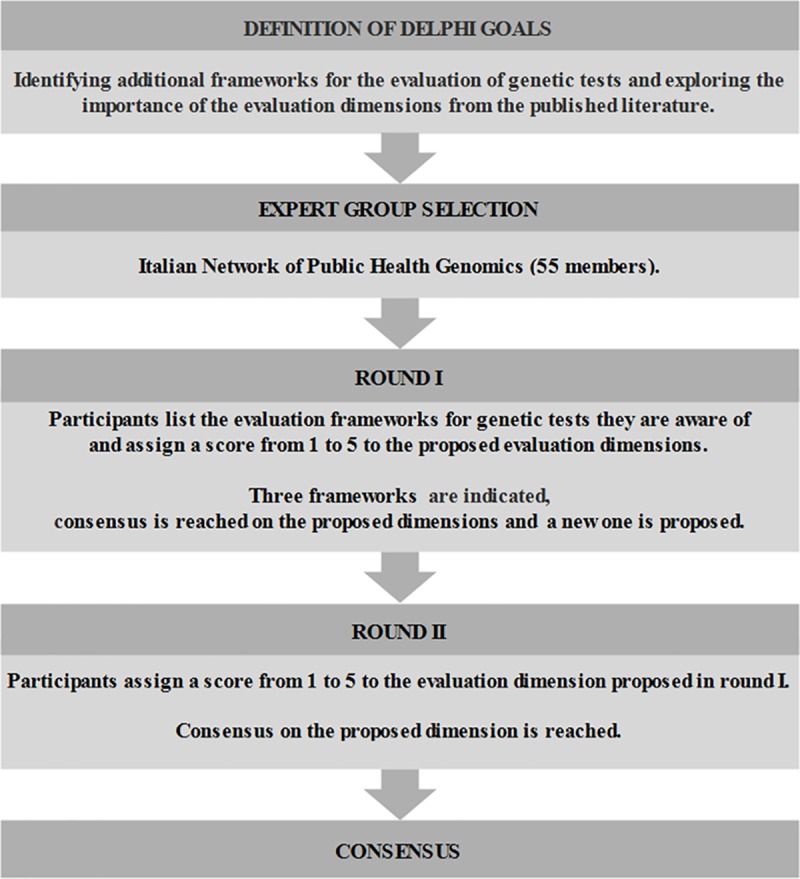
Steps of the Delphi survey.

The online Delphi survey involved 55 members of the Italian Network of Public Health Genomics (GENISAP) (Fig 1) [7]. The network includes clinicians, epidemiologists, biologists, counselors, and health care managers who work at different levels in the field of human genetics and public health (Table 1). The study did not require ethical approval because it used a non-sensitive and anonymous questionnaire administered to experts. Participants were informed about the study during a face-to-face meeting, followed by individual phone calls. In particular, it was explained that answering and sending back the questionnaire would constitute consent to participate in the study.

**Table 1:** Characteristics of GENISAP experts.

Experts (total = 55)		
Age (years), average, range	52.6	31–77
Gender, *n*, % of total		
Male	24	43.6
Female	31	56.4
Region, *n*, % of total		
Northern Italy	20	36.4
Central Italy	30	54.5
Southern Italy and Islands	5	9.1
Degree, *n*, % of total		
Medicine	34	61.8
Biology	20	36.4
Economics	1	1.8
Primary employment, *n*, % of total		
University	32	58.2
Healthcare institution	18	32.7
Government institution	5	9.1

#### Round I

We developed a two-part survey that includes both open- and closed-ended questions. The questionnaire is shown in S2 File. In the first part, we asked the experts to list the evaluation frameworks they were aware of. In the second part, we asked them to rate, on a five-point scale ranging from 1 (minimal importance) to 5 (utmost importance), the following dimensions, identified from the preliminary results of the systematic review: overview of the disease and the test under study; analytic validity; clinical validity; clinical utility; ethical, legal and social implications (ELSI); delivery models; organizational aspects; economic evaluation. Each dimension was provided with a definition in order to standardize the interpretation. Where, for a particular dimension, at least 65% of panelists agreed, we considered that a consensus had been reached. In addition to the rating, the experts had the option of providing comments and extra dimensions. The survey was piloted on a convenience sample of six academicians known to the research team. To increase the response rate, we contacted the experts by phone before emailing the definitive survey. They had three weeks to answer, during which time two remainders were e-mailed.

#### Round II

Since the threshold of 65% had already been exceeded in the first round for all the proposed evaluation dimensions, we used round II to evaluate one new dimension proposed by some experts in round I. Therefore, after communicating the results of round I to the panelists, we asked them to assign a score from 1 to 5 to the new dimension, which had also been provided with a definition. As in round I, they had three weeks to answer, were given the chance to provide comments and add dimensions and were encouraged by phone and e-mail remainders. At the end of the second round, having reached a satisfactory level of agreement (i.e. ≥65%) among the experts, we terminated the consensus procedure and communicated the results to participants.

### Framework development

The framework was built on the evaluation dimensions identified through the systematic review and the Delphi procedure and refined in frequent meetings in the presence of the moderator (P.V.). The analysis of the strengths and weaknesses of the existing frameworks guided the whole process. The final version of the framework was sent to the Delphi panelists, and their feedback was collected via e-mail.

## Results

### Systematic review

The systematic search identified 29 frameworks published between 2000 and 2017, mainly in the USA and Europe [3]. They are mostly based on the ACCE Framework (n  =  13 models), or on the HTA process (n  =  6), or both (n  =  2). The remaining refer to the Wilson and Jungner screening criteria (n  =  3) or to a mixture of different preexisting frameworks (n  =  5). The ACCE framework is therefore the most popular approach to the evaluation of genetic tests, followed by the HTA process. The evaluation dimensions retrieved were: test and clinical condition overview, analytic validity, clinical validity, clinical utility, ELSI, delivery models, organizational aspects, economic evaluation and patient perspective (the last of these was not retrieved in the preliminary results and therefore was not included in the Delphi survey). Due to the spread of the ACCE model, the most frequently employed dimensions were analytic validity, clinical validity, clinical utility and ELSI. An economic evaluation was always proposed but rather superficially. The delivery models and organizational aspects were frequently missing in the retrieved evaluation frameworks and any analysis of these aspects, mostly represented by the HTA-based evaluation frameworks, was usually not well structured. Finally, the direct experience of patients has rarely been considered as an independent evaluation dimension. The most frequently used format was the key questions format; about half of all frameworks gave suggestions on the process of evidence review; and only five frameworks provided criteria for deciding on the use of the test based on the evidence collected. Since decision makers are the main audience for which the evaluation process is intended, the lack of attention to context-related aspects of testing (delivery models, economic evaluation, organizational aspects) and to the decision-making process are arguably the main limitations of the retrieved frameworks [3]. Finally, even where these frameworks were mainly developed to address single-gene testing, some, such as the NHS UKGTN Gene Dossier and the Clinical Utility Gene Card [9, 10], have also proved effective for applications based on next-generation sequencing.

### Delphi survey

Of the 55 experts invited to participate in round 1, 43 completed the questionnaire, a response rate of 78%. Three frameworks for the evaluation of genetic tests, already included in our systematic review, were suggested by these experts [4, 11, 12], together with one non-genetic test-specific framework, the EUnetHTA core model [13]. Each dimension was ranked by at least 42 participants. The consensus was very strong (Table 2): 82% of respondents scored all dimensions 4 or 5. In line with the findings from the literature, the dimensions of the ACCE framework were the most highly regarded: *overview of the disease and the test under study*, *analytic validity*, *clinical validity*, *clinical utility* and *ELSI* were all scored 4–5 by at least 95% of respondents, followed by *economic evaluation*, which 93% of respondents scored 4–5. On the other hand, *delivery models*, *organizational aspects* and *research priorities* were scored 4–5 by less than 90% of experts, respectively 89%, 88% and 82%. Relevant comments were raised about some dimensions (Table 3). For instance, it was proposed that the potential multidimensionality of clinical utility should be addressed by defining the core standards used in its evaluation. The importance of considering the economic impact of different delivery options for the test under assessment, and the need for transparency of economic evidence reporting, were both underlined. Finally, a new dimension, the patient perspective, was suggested (anticipating the final results of the systematic review). Of the 43 experts who participated in round 1, 33 completed round 2, a repeat response rate of 76%. The patient perspective was ranked by all participants and was scored 4–5 by 67%.

**Table 2:** Delphi consensus procedure: Score assigned to the proposed evaluation dimensions.

Evaluation dimension	Score ^a^ (% of respondents)					
	1	2	3	4	5	NR
Test and clinical condition overview	0	0	2	14	84	0
Analytic validity	0	0	2	0	98	0
Clinical validity	0	0	2	7	89	2
Clinical utility	0	0	2	5	91	2
ELSI	0	0	3	23	72	2
Economic evaluation	0	0	7	42	51	0
Delivery models	0	0	9	28	61	2
Organizational aspects	0	0	12	39	49	0
Patient perspective	6	3	24	34	33	0

^a^ Score: 1 = of little importance; 5 = of great importance

Abbreviations: ELSI, Ethical, Legal and Social Implications; NR, No Response

**Table 3:** Delphi consensus procedure: Most relevant comments on the proposed evaluation dimensions.

Evaluation dimension	Most relevant comments from the respondents
Clinical utility	There is no agreement on which outcomes should be measured under the umbrella of clinical utility (i.e. should it include personal and social aspects, as well as clinical?). This issue therefore needs clarification.
Economic evaluation	The economic impact of different delivery options for the genetic test under assessment should be considered. Moreover, to contextualize the results of the existing economic analyses, methodological aspects should be taken into account.
Organizational aspects	Considering and contextualizing organizational aspects is very important, especially for publicly funded healthcare systems.
Delivery models	Important but difficult to assess, especially in the case of common complex diseases

In summary, the Delphi survey confirmed the importance of the ACCE dimensions in assessing the technical and clinical value of a genetic test, as had already emerged from the systematic review. Nonetheless, the experts appeared to be inclined to include the context-related aspects of testing in the evaluation process.

### Framework

#### General structure

We created a framework that integrates the two most popular approaches to the evaluation of genetic tests i.e. the ACCE model, which is well-suited to the assessment of the technical and clinical value of genetic tests, and the HTA approach, which analyzes in a more systematic manner the organizational and delivery aspects of genetic services. As a reference tool for the HTA approach we adopted the EUnetHTA core model, well recognized at a European level and supported by the results of the Delphi survey [13]. The final version of the framework, as approved by the GENISAP experts, is fully described in the handbook attached as S1 File. It consists of four sections (Table 4). The first two sections guide the process of evidence collection for, respectively, the genetic test itself and its delivery models. Each section comprises an introduction (“*Test and clinical condition overview”* and *“Delivery models overview*”) followed by the actual evaluation dimensions. Four dimensions refer to the “genetic test” (*analytic validity; clinical validity; clinical utility; personal utility*) and four to its “delivery models” (*organizational aspects; economic evaluation; ELSI; patient perspective*). For each dimension, suggestions on which sources of evidence to consult are provided. The third section deals with evidence gaps unearthed during the collection of evidence. Finally, the fourth section suggests three criteria to summarize the collected evidence into practical points for decision.

**Table 4:** The proposed evaluation framework.

***I*. *GENETIC TEST***	**TEST AND CLINICAL CONDITION OVERVIEW** CLINICAL CONDITION Clinical presentation and pathophysiology Genetic background Public health impact GENETIC TEST General features Technical features Clinical context **ANALYTIC VALIDITY** ANALYTIC SENSITIVITY ANALYTIC SPECIFICITY ACCURACY PRECISION ROBUSTNESS LABORATORY QUALITY CONTROL **CLINICAL VALIDITY** SCIENTIFIC VALIDITY TEST PERFORMANCE Clinical sensitivity and specificity Positive and negative predictive value Modifiers **CLINICAL UTILITY** AVAILABLE INTERVENTIONS EFFICACY EFFECTIVENESS SAFETY **PERSONAL UTILITY**
***II*. *DELIVERY MODELS***	**DELIVERY MODELS OVERVIEW** HEALTHCARE PROGRAMS LEVEL OF CARE PATIENT PATHWAY **ORGANIZATIONAL ASPECTS** EXPECTED DEMAND RESOURCES MANAGEMENT OTHER ORGANIZATIONAL REQUIREMENTS Education of professionals, patients and citizens Information dissemination to professionals, patients and citizens Cooperation, communication and coordination Quality assurance, monitoring and control BARRIERS TO IMPLEMENTATION **ECONOMIC EVALUATION** **ETHICAL, LEGAL AND SOCIAL IMPLICATIONS** **PATIENT PERSPECTIVE**
***III*. *RESEARCH PRIORITIES***	**EVIDENCE GAPS & RESEARCH PRIORITIES**
***IV*. *DECISION POINTS***	**NET BENEFIT** **COST-EFFECTIVENESS** **FEASIBILITY**

#### Section I—The genetic test

Section I starts with an *introduction* aimed at defining the clinical condition and the test under examination. The clinical condition should be characterized in terms of clinical presentation, pathophysiology, genetic background, and public health impact. The genetic test should be generally described, reporting the identifiable genes and variants; technically defined, with a focus on the analytic procedure; and clinically contextualized in terms of purpose, target population and actual use. This introduction is the foundation for the study of the related dimensions, i.e. analytic validity, clinical validity, clinical utility and personal utility. *Analytic validity* assesses the accuracy with which a particular genetic characteristic, such as DNA sequence variants, chromosomal deletions, or biochemical indicators, is identified in a given laboratory test [14]. It is a function of many factors of which the most commonly considered are analytic sensitivity and specificity, accuracy, precision, robustness and laboratory quality control [4, 15, 16]. *Clinical validity* assesses the ability of the test to accurately and reliably detect or predict a clinical condition [17]. It consists of two parts: the assessment of scientific validity, which is the evidence for gene-disease association; and the assessment of test performance in terms of sensitivity, specificity, positive and negative predictive value, and influencing factors [18]. *Clinical utility* assesses the health impact of the test in terms of risks and benefits; it considers the availability, efficacy, effectiveness and safety of the interventions to be put in place, depending on the test results, compared to the current practice [14, 19]. Finally, *personal utility* considers the full range of personal reasons for testing and the personal effect of testing, both of which are subjective and non-health related (or indirectly health related), e.g. improving the patient’s understanding of the disease, enabling reproductive and life planning, etc. [20, 21].

#### Section II—Delivery models

Section II starts with an *introduction* aimed at defining the delivery models for the provision of the test under study. A delivery model is the broad context in which genetic tests are offered to individuals and families with or at risk of genetic disorders [22]. It should be explained by three elements: the healthcare program, i.e. the set of interventions preceding, following and including the genetic test, for a specific target population and with a specific health purpose [23, 24]; the level of care (e.g. primary or specialist care level) in which the provision of the genetic healthcare program is integrated and coordinated [25]; and the patient pathway, i.e. the patient flow through different professionals from the point of access to the genetic test to the diagnosis and treatment of the genetic disorder [26]. The description of the delivery model is essential for studying the related dimensions, i.e. organizational aspects, economic evaluation, ELSI and patient perspective. Analysis of *organizational aspects* leads to an estimate of the expected demand for the genetic test under study and the resources needed to implement the related healthcare program. It should also consider possible barriers to implementation and further requirements such as education and training of staff; information dissemination to professionals, patients and citizens; access to care; cooperation, communication and coordination between and within organizations; quality assurance, monitoring and control systems [13]. The *economic* dimension assesses the quantity and quality of cost-effectiveness and cost-utility evidence for alternative genetic testing programs [27]. The *ELSI* dimension considers an ensemble of heterogeneous concepts, namely the moral value that society confers on the proposed interventions, the specific related norms and the impact on the patient’s and his or her family’s social life [16]. Some of the most common issues to address are: autonomy; equity; discrimination; privacy; confidentiality; informed consent; societal values; psychosocial impact; and reproductive issues [28, 29]. The *patient perspective* dimension collects qualitative and quantitative evidence on the perspective of patients who, as the direct beneficiaries of the genetic program in question, can contribute to an understanding of its value (N.B. the term patient is used to define any user of a genetic service) [30].

#### Section III

Section III was designed to address the evidence gaps that will almost certainly be unearthed during the evaluation process. These gaps, other than in the quantity and quality of the evidence, may also relate to evidence generalizability and relevance to the specific context of evaluation. In this section it should be defined whether the amount of evidence for each dimension is exhaustive, incomplete or missing (Table A in S1 File). In the last two cases, the evidence gaps should be formulated as specific research questions, in order to steer future research and inform decision makers about the real state of the art.

#### Section IV

Section IV suggests three criteria for summarizing the evidence collected and supporting the decision-making process on the use of the test: the net benefit, cost-effectiveness and feasibility of the testing program.

The net benefit of an intervention is the balance between its benefits and harms. To justify an intervention, benefits should adequately exceed harms. The dimensions to be considered when assessing the net benefit of a testing program are: analytic validity, clinical validity, clinical utility, personal utility, ELSI, and patient perspective. Based on these dimensions, the net benefit can be assigned to one of four classes (A–D), ranging in descending order from positive to negative (Table B in S1 File).

The analysis of the cost-effectiveness of an intervention should help decision makers understand whether they are satisfied that the additional healthcare resources, required to make the test available to those who could benefit from it, should be spent on the intervention rather than on something else [31]. Four classes (A-D) of cost-effectiveness can be defined, ranging in descending order from highly cost-effective to not cost-effective (Table C in S1 File).

The feasibility of genetic testing programs is determined from the organizational analysis. It can be defined as the probability of overcoming the identified barriers to implementation and can be rated in three classes (I–III) in descending order from easy to impossible (Table D in S1 File).

When considering net benefit and cost-effectiveness, decision makers should be given the opportunity to take into account the quality of the supporting evidence. Specifically, the quality of the evidence should be assessed using validated instruments and a minus or plus sign (indicating low or high quality, respectively) should be added to each rating class, as appropriate.

The approach suggested by Section IV is not mandatory and the three criteria should be used at the discretion of decision makers.

## Discussion

We have developed a new framework aimed at producing technology assessments on which decision makers, i.e. the Ministry of Health with the support of the National Agency for Regional Healthcare Services and the National Institute of Health in Italy, can base national provision and coverage decisions regarding genetic and genomic technologies.

The framework is distinguished by a dual focus on both the technology and its delivery models. This is consistent with the HTA evolution process theorized by Battista, according to which HTA has evolved through three distinct phases: the “machine phase”, focusing on the technical performance of health technologies; the “clinical outcomes phase”, focusing on outcomes of technology use in clinical settings; and the “delivery modes phase”, focusing on how health services are organized and delivered [5]. While the first two phases are already well represented in the ACCE framework, the third phase needs more consideration. We think that our framework, which develops the delivery models phase, would bring the evaluation process closer to the context of implementation of genetic technologies, and thus help decision makers to secure an efficient and equitable allocation of health care resources and services.

The first section of the framework, mainly based on the ACCE model, guides the assessment of the genetic test. In particular, by adopting the dimensions of the ACCE model, this section addresses some aspects of genetic tests that are fundamental to an understanding of their clinical suitability, such as analytic and clinical validity, which are not adequately addressed by standard methods of technology assessment. Because analytic validity cannot be defined by a universal set of criteria, applicable to all kinds of test, we suggest looking at the criteria most often addressed in the current literature [4, 14, 15]. As recommended by Burke & Zimmern, we assessed separately two distinct properties of clinical validity: the widely cited clinical test and the less reported gene-disease association [17]. While in some cases clinical utility includes individual, familial, and societal benefits [32], we defined the essential evaluation standards for clinical utility in their narrowest sense, i.e. the improvement in health outcomes due to the test and the subsequent clinical interventions. We agree with Grosse & Khoury that clinical utility should be measured using objective metrics and that health outcomes are the most critical factor in setting priorities for public health, especially in publicly funded healthcare systems [18]. Nevertheless, we understand that subjective, familial or societal benefits cannot be ignored. That is why we introduced the dimension of personal utility in this first section and the ELSI dimension in section two. However, the assessment of these two dimensions presents at least two challenges: establishing their weight in the overall assessment of a genetic test [19, 32]; and defining the most useful and relevant criteria for exploring them, given the heterogeneity of genetic tests and socio-cultural backgrounds. The work of Khoeler et al. might help with personal utility, as they made an empirical effort to delineate its relevant elements such as self-knowledge, knowledge of the condition, coping, and life planning [20, 33]. Our framework asks for a report on the most important and common considerations of personal utility, even if only in qualitative terms, as they could help decision makers understand the whole picture. Taking the example of predictive testing for *BRCA1/2* mutations, personal aspects to be considered might be the shift of cancer risk perception, the personal meaning ascribed to the mutation status, and the experience of being a mutation-negative/positive individual, with regard to the role of family dynamics [34, 35]. For instance, two qualitative studies point out that family dynamics might challenge both mutation-positive and -negative women, giving rise to psychosocial needs that should be addressed by healthcare professionals [34, 35].

The second section of the framework, mainly based on the HTA approach, focuses on the delivery models. When collecting economic, organizational, ELSI and patient perspective evidence, a consideration of delivery models could be very valuable for decision makers. For example, BRCA1/2 genetic tests could be delivered in a population screening program, where healthy women invited for mammography may be advised to take a BRCA1/2 test on the basis of their family history. In this case, local healthcare units would need to coordinate active call of patients, risk assessments, genetic counseling, preventive or treatment pathways, cascade screening etc. A BRCA1/2 genetic test could also be delivered in a specialist care setting, such as an oncology clinic, where women with breast cancer may be advised to take the test on the basis of their personal and family history. In this case, active call to recruit the general population is not needed, and counseling and other interventions will probably take place in a specialist context. These two different delivery models will definitely have different costs, benefits, organizational issues, ethical concerns and impact on patient perspective. Thus, they will prove more or less appealing for decision makers. This is why shifting the focus from the genetic test to its delivery models is fundamental when considering implementation questions. While some existing frameworks include economic or organizational considerations within clinical utility (e.g. the ACCE framework), we believe that treating these dimensions independently could provide clearer guidance to evaluators and a more useful represention of the evidence collected to decision makers. Regarding economic implications, our framework asks that not only are the results of the existing economic analyses reported, but also their methodological details (e.g. comparator, perspective, sensitivity analysis). In this way decision makers can understand the quality of the analysis performed and its relevance to their own context [13]. Contextualization also affects organizational analysis, as it cannot but follow the level at which decisions are taken, e.g. regional, departmental, hospital. Moreover, the multiplicity of objectives and assessment criteria makes this analysis less pre-determined and more complicated than others [13]. ELSI analysis faces the same challenge already described for personal utility. In this case, help may come from the work of the US Genetic Alliance and Institute of Medicine, which delineated the major concerns that should be addressed, such as equity, discrimination and confidentiality [28, 29]. Moreover, it is necessary to consider that ELSI are often interrelated, both within the ELSI dimension and with other evaluation dimensions. A recent paper identified the interrelationships among more than 80 ELSI, combining them into five clusters: patient preferences and experiences (e.g. patient-physician relationship, social stigma, consequences for family members, etc.); patient quality of life and function (e.g. effect on daily, social activities and social function, etc.); patient burden/harm (e.g. side effects and adverse events; risk of incorrect test results, etc.); fairness (e.g. universal access; confidentiality; discrimination, etc.); and organizational (e.g. informed consent; continuum of patient care; privacy, etc.) [36]. Finally, despite the increasing emphasis on the need for more patient-centered evaluations, a set of criteria for assessing the patient perspective is also lacking [37]. Our framework requires that evidence on the patient perspective be derived from both quantitative and qualitative studies, e.g. surveys or interviews. This is the only way to learn about the experiences, attitudes, beliefs and expectations of living with an illness and using a genetic service.

Section three addresses one of the major difficulties encountered in the evaluation of genetic tests, i.e. the collection of evidence. Finding the answer to some of the evaluation questions, especially those related to the clinical value and to the implementation of a genetic test, is not a simple matter. In fact, while gene discovery studies are carried out relatively rapidly, translation studies (including randomized clinical trials and appropriately conducted observational studies) often lag behind [6, 38]. Moreover, the lack of evidence on effectiveness also affects the evaluation of cost-effectiveness [39].

Section four of the framework aims to synthesize the collected evidence into practical points for decision. We think that it is critical for decision makers to consider the net benefit, cost-effectiveness and feasibility of a particular test, as well as the quality of scientific evidence supporting it. Nevertheless, defining an appraisal framework is beyond the scope of our work. In this regard, it should be made clear that our framework only covers the process of evidence collection. This is undoubtedly the main focus of technology assessment, but it is only one step of an ideal decision pathway. Evidence collection should be preceded by a priority setting phase, as the number of available genetic tests far outweighs the resource available for their assessment, and followed by an appraisal phase, which provides recommendations on the use of a test by prioritising the various elements of the large body of evidence collected.

If the comprehensiveness of the evaluation dimensions is the strength of our framework, it is also the main limitation. In fact, the considerable time needed to complete the evaluation process clashes with the rapid development and dissemination of genetic tests. Moreover, the lack of direct evidence for several dimensions would make it necessary to refer to grey literature, resulting in a higher probability of error and a reduced quality of evidence. Finally, decision makers might be hampered by the large amount of information collected (and therein lies the utility of section four). Another limitation of our work might be the intention to use a single framework to evaluate different types of test, but we do not claim the framework to be a universal guide. It is a wayfinder that can be adapted to the individual test under study, for which particular dimensions and criteria might be appropriate, optional or not applicable. We chose a handbook format to ensure flexibility, it being less restrictive than a questionnaire or checklist. On the other hand, we believe that the adoption of a single framework for the evaluation of different types of test might allow comparison of technologies and the sharing of information. Moreover, our framework could be of benefit for the assessment of other types of technologies and services. In fact, by bringing together the ACCE model with the HTA approach, we have created a framework that adopts genetic test-specific and widely recognized dimensions and terminology, but is conceptually and structurally similar to generic technology assessments. Finally, two main concerns result from the selected Delphi survey sample, i.e. the GENISAP experts. The first one is the representativeness of the sample. In fact, while the primary audience of our framework is represented by Italian national and local government decision makers, the GENISAP sample comprises predominantly academics. Nevertheless, in Italy academics are often consulted for healthcare decision making, and are particularly involved in the evidence collection for decision making. For this reason, we think that the contribution of the GENISAP experts was extremely useful for the development of the framework, given also their specific experience in the field of public health genomics. The second concern is the generalizability of our framework to other countries. In fact, it was developed with the contribution of Italian experts only. Nevertheless, it is also based on information derived from a systematic international literature review, so we believe it may be a suitable model for other health systems.

Even though the final version of the framework was approved by the GENISAP experts, to gain first-hand experience we are currently validating it by assessing the *BRCA1/2* genetic test for susceptibility to breast and ovarian cancer. The framework seems to be an efficient tool for collecting and organizing existing evidence, although it takes some time to complete. Moreover, as anticipated in our previous work [23], the usefulness of considering both the genetic test and its delivery model is confirmed. Nevertheless, we are aware that our framework must be considered “under development” until it has been definitively validated.

Our work was financed by the Italian Ministry of Health to implement the 2013 National Plan for Public Health Genomics, one of the few examples of a national policy in Europe aimed at translating genomics into clinical practice at a central level [40]. A further step has recently been made with the approval of the National Plan for Innovation of the Health System based on omics sciences [41]. It outlines the ways in which innovations in the omics field should benefit the National Health System in the areas of prevention, diagnosis and care, taking into account effectiveness and sustainability [41].

In conclusion, our framework aims to broaden the evaluation of genetic and genomic tests to include service delivery. While the context dependence of this analysis reduces its transferability, it is the only way to meet the specific requirements of decision makers. Italian experts gave positive feedback, but the framework will probably require further refinement. Subsequently, regular updates will be needed to keep pace with new innovations in the field.

## Supporting information

S1 File Evaluation framework handbook. (PDF)

S2 File Delphi survey questionnaire. (PDF)

S3 File Delphi survey dataset. (XLS)
