# Supplementary material for: A proposal of a new evaluation framework towards implementation of genetic tests
Source: PLoS One. 2019 Aug 5;14(8):e0219755. doi: 10.1371/journal.pone.0219755 (PMC6681956; doi:10.1371/journal.pone.0219755)
Supplement: S1 File — (PDF) [file pone.0219755.s001.pdf]

SUPPORTING INFORMATION 1

EVALUATION FRAMEWORK HANDBOOK

Table of contents

1. GENETIC TEST p. 1

1.1 TEST AND CLINICAL CONDITION OVERVIEW p. 1

1.2 ANALYTIC VALIDITY p. 2

1.3 CLINICAL VALIDITY p. 2

1.4 CLINICAL UTILITY p. 3

1.5 PERSONAL UTILITY p. 4

2. DELIVERY MODELS p. 5

2.1 DELIVERY MODELS OVERVIEW p. 5

2.2 ORGANIZATIONAL ASPECTS p. 6

2.3 ECONOMIC EVALUATION p. 6

2.4 ETHICAL, LEGAL AND SOCIAL IMPLICATIONS p. 7

2.5 PATIENT PERSPECTIVE p. 8

3. RESEARCH PRIORITIES p. 9

4. DECISION POINTS p. 10

4.1 NET BENEFIT p. 10

4.2 COST-EFFECTIVENESS p. 11

4.3 FEASIBILITY p. 12

REFERENCES p. 12

SECTION 1 – GENETIC TEST

1.1 TEST AND CLINICAL CONDITION OVERVIEW

This section aims at introducing the clinical condition and the genetic test under examination with a brief description.

Clinical condition: the starting point for considering the use of a genetic test is a well-defined clinical condition, for which the test is expected to improve care [1]. The clinical condition should be characterized in terms of clinical presentation and pathophysiology; genetic background; and public health impact [2–5].

Clinical presentation and pathophysiology: the main information about the etiopathogenesis and the natural history of the related condition should be reported, as well as its clinical features. Moreover, the current clinical management should be described by reference to the existing guidelines. In the case of a pharmacogenetics test, the main information regarding the drug should be summarized (e.g. drug name, category, and indications; pharmacodynamics and pharmacokinetics; method of administration; adverse effects; response assessment parameters).
Genetic background: the main information about the genetic background of the described condition should be reported, such as the genes, genomic or chromosomic regions involved. Moreover, the known variants and their frequency should be reported by reference to specific databases. Attention should be payed to the patterns of inheritance, the penetrance and the potential modifiers of the genotype-phenotype correlation. In the case of a pharmacogenetics test, the association genotype-phenotype-expected effects should be clearly described.
Public health impact: the main information about the health impact of the described condition should be reported. Epidemiological features should be described (i.e., incidence and prevalence; health relevance in terms of mortality, morbidity and other expected outcomes for the patient, its family and the society). When available, the associated costs should be reported, using a healthcare or third-payer perspective (e.g. direct costs of health care system, health insurances, co-payment). Finally, unmet needs regarding the condition in question should be underlined.

Genetic test: the main general and technical features of the genetic test under assessment should be reported. Moreover, the clinical context of testing use should be outlined.

General features: general features of the genetic test should be reported, such as type of test (diagnostic; carrier; preclinical or presymptomatic; susceptibility, pharmacogenetics, etc.); identifiable genes and variants; manufacturers.
Technical Features: technical features related to the analytical methods should be reported.
Clinical context: the clinical context of testing use should be described in terms of purpose, target population, and use. Defining the clinical purpose of the test is necessary to determine its intended benefit, such as reducing mortality or adverse effects [6]. The target population should be described reporting age, gender, geographical and genealogical origin and eligibility criteria for testing. Then, information about target population size, condition prevalence and variants frequency should be collected. Where appropriate, the actual use of the test should be described (e.g. number of test provided annually, existing guidelines,

regulatory status, etc.) Finally, it should also be specified whether the genetic test is intended to replace or supplement another technology in the management chain [7].

SOURCES OF EVIDENCE: reviews (systematic reviews and meta-analyses, narrative reviews); HTA reports; guidelines; text books; observational epidemiologic studies (cohort studies, case-control studies, transversal studies, descriptive studies); qualitative research; national and international reports, registers and statistics; administrative databases (e.g. DRG); genetic variants databases; regulatory acts or programming documents (national, regional or local); medicine agencies data (e.g. Food and Drug Administration); clinical laboratory or manufacturer data; consensus conference; experts opinion.

1.2 ANALYTIC VALIDITY

Analytic validity refers to the accuracy with which a particular genetic characteristic, such as a DNA sequence variant, chromosomal deletion, or biochemical indicator, is identified in a given laboratory test [8]. It assesses test performance in the laboratory as opposed to the clinic and includes different elements depending on the type of test under evaluation [9]. The most commonly considered are: analytic sensitivity, analytic specificity, accuracy, precision, robustness and laboratory quality control [2,9–11].

Analytic sensitivity: how effectively a test can detect all true positive specimens, as determined by a reference method [12].

Analytic specificity: the ability of a measurement procedure to measure solely the analyte of interest [12].

Accuracy: the closeness of agreement between a test result and true value of what is being measured [12].

Precision: the closeness of agreement between independent results of measurements obtained under stipulated conditions [12].

Robustness: the ability of a method to remain unaffected by small fluctuations in assay parameters; it is often assessed through inter-laboratory comparison studies or by varying parameters such as temperature and relative humidity to determine the operating range of the method [12].

Laboratory quality control: assesses the procedures for ensuring that results fall within specified limits [11].

SOURCES OF EVIDENCE: reviews (systematic reviews and meta-analyses, narrative reviews); HTA reports; collaborative multicentric studies; external proficiency testing schemes and interlaboratory comparison programs; validation studies and method comparisons; medicine agencies data (e.g. Food and Drug Administration); clinical laboratory or manufacturer data [13].

1.3 CLINICAL VALIDITY

The clinical validity dimension assesses the ability of the test to accurately and reliably detect or predict a clinical condition [9]. It consists of two parts: the assessment of scientific validity and the assessment of test performance [3,9,14].

Scientific validity: the evidence of biomarker-disease association. It is essentially a matter for epidemiological studies which are normally carried out by the scientific community [14].

Test performance: the degree to which variants can distinguish between those who will develop a clinical condition from those who will not. It is usually described in terms of sensitivity, specificity, positive and negative predictive value and influencing factors (condition prevalence, condition penetrance and genetic-environmental modifiers) [8,11].

Clinical sensitivity: the proportion of individuals for whom the test result correctly identifies or predicts the presence of a well-defined disorder [12].
Clinical specificity: the proportion of individuals for whom the test result correctly detects or predicts the absence of a well-defined clinical disorder [12].
Positive and negative clinical predictive value: the probabilities that people (within a defined population) with positive test results will get the disease (positive predictive value) and that people (within a defined population) with negative results will not get the disease (negative predictive value) [12].
Influencing factors:

- *Penetrance:* the relationship between genotype and phenotype. It is the probability or likelihood that the condition (or phenotype) will be expressed when a particular genotype is present [12].
- *Prevalence:* the proportion of individuals in the selected setting or population who have the phenotype [12].
- *Modifiers:* other genetic or environmental factors that may interact with the genetic alteration being studied and the outcome of interest. Modifiers can affect expressivity, which refers to the variability of signs or symptoms that occur with a phenotype [12].

SOURCES OF EVIDENCE: reviews (systematic reviews and meta-analyses, narrative reviews); HTA reports; guidelines; observational epidemiologic studies (cohort studies, case-control studies, cross-sectional studies, descriptive studies); clinical laboratory or manufacturer data; consensus conference; expert opinion.

1.4 CLINICAL UTILITY

Clinical utility refers to the health impact of a genetic test in terms of risk and benefit, compared to the current practice [15]. Specifically, it measures the improvement in health outcomes - such as mortality, morbidity, or disability – due to the interventions adopted on the base of test results and the risks that might occur [8,16]. The criteria to be considered are therefore: available of interventions; efficacy; effectiveness; and safety.

Available interventions: interventions to be put in place depending on the test results.

Efficacy: the ability of the test to bring about the intended purpose when used under the most favorable circumstances [3]**.**

Effectiveness: the degree to which attainable objectives are in fact attained under routine conditions [3].

Safety: umbrella term for any unwanted or harmful effects caused by using a health technology; it includes the description of harms, susceptibility patient group, and risk management [7].

Harms: this section describes kind, incidence, severity and duration, relation to dosage and frequency of applying the technology as well as consequences of false positive, false negative and incidental findings [7].
Susceptible patient groups: this section describes patient group that are more likely to be harmed through use of the technology [7].
Risk management: this section describes the requirement for specific training, use of protocols or available guidelines which may reduce the occurrence or severity of the harm [7].

SOURCES OF EVIDENCE: reviews (systematic reviews and meta-analyses, narrative reviews); HTA reports; guidelines; clinical trials (with or without randomization); observational studies (cohort studies, case-control studies, cross sectional studies, descriptive studies); decision modeling; pilot studies; surveys; national and international reports, registry and statistics; clinical laboratory or manufacturer data; consensus conference; expert opinion.

1.5 PERSONAL UTILITY

Personal utility is a broad category that includes the full range of personal reasons for testing and the personal effect of testing, both of which are subjective and non-health related (or indirectly health related) [17,18]. Different from clinical utility, personal utility does not directly lead to improve health outcomes but has an effect on the overall well-being of individuals [18].

Considerations on personal utility should be reported, even if only in qualitative terms, in order to give a more complete picture of the value of a genetic test. This evaluation can be guided by the work of Kohler et al. They made an empirical effort to delineate relevant elements of personal utility such as self-knowledge, knowledge of the condition, coping, family dynamics, and reproductive and life planning [18,19].

SOURCES OF EVIDENCE: reviews (systematic reviews and meta-analyses, narrative reviews); HTA reports; clinical trials (with or without randomization); observational studies (cohort studies, case-control studies, cross sectional studies, descriptive studies); pilot studies; surveys; qualitative research; patient's organizations documents.

SECTION 2– DELIVERY MODELS

2.1 DELIVERY MODELS OVERVIEW

A delivery model is the broad context in which genetic tests are offered to individuals and families with or at risk of genetic disorders [20]. To describe a delivery model, it is necessary to shift the focus of the evaluation from the genetic test to the healthcare program in which the test is provided. Then, the healthcare program should be contextualized in terms of patient's pathway and level of care [20].

Healthcare program: any type of health intervention that includes a genetic test in a target population with a specific health purpose [21,22]. Given that a genetic test could be offered in different health care programs, the one under assessment should be describe using the following key elements (some of which have been already defined in Section I):

- *Target population*
- *Health purpose*
- *Health intervention*

  - Genetic test.
  - Any activity preceding the genetic test (e.g. genetic counseling, risk assessment, etc.).
  - Any activity following the genetic test (treatment/prevention strategy).

Level of care: the level of care in which the provision of the genetic healthcare program is integrated and coordinated. Within the healthcare system, the possible levels are: genetic specialty; other specialties; primary care; and screening programs [23]. Outside the healthcare system there is the opportunity of direct to consumer. Therefore, the level of care should be described from one of the following:

- *Genetic specialty:* genetic program integrated in genetic centers.
- *Other medical specialties:* genetic program integrated in other medical specialties (e.g. oncogenetics, neurogenetics, cardiogenetics).
- *Primary care:* genetic program integrated into primary care.
- *Screening programs:* genetic program provided in screening programs (e.g. prenatal and newborn screening).
- *Direct to consumer:* genetic test ordered directly by an individual, without involving healthcare professionals.

Patient's pathway: Even though the healthcare program is integrated in one of the level mentioned above, the patient may meet different professionals. The patients' pathway through different professionals, from the point of access to the genetic test to the diagnosis and treatment of the genetic disorder, should be described. For example Gu et al. identified the following four pathways [24]:

- *Patient–Doctor (general practitioner/specialist)–Counselor*
- *Patient–Doctor (general practitioner/specialist)–Lab*

- *Patient–Counselor–Lab*
- *Patient–Commercial lab (i.e., direct-to consumer genetic testing).*

SOURCES OF EVIDENCE: reviews (systematic reviews and meta-analyses, narrative reviews); HTA reports; guidelines; pilot studies; best practices; own studies (e.g. questionnaires and interviews of different actors); regulatory acts or programming documents (national, regional or local); reports and documents of hospital and hospital districts; consensus conferences; expert opinion [7].

2.2 ORGANIZATIONAL ASPECTS

The analysis of organizational aspects estimates the expected demand for the genetic test under study and the resources needed to implement the related healthcare program; it should also consider possible barriers to implementation and further requirements [7].

Expected demand: an estimate of the need for the test under assessment according to the disease/mutation prevalence in the target population [25,26].

Resources management: an estimate of the human, material and economic resources needed to implement the healthcare program, of their availability and opportunity to be acquired [7].

Other organizational requirements: other organizational requirements such as [2,7,27]:

- *Education of professionals, patients and citizens*
- *Information dissemination to professionals, patients and citizens*
- *Process ensuring access to care (special populations, remote areas, rare diseases)*
- *Cooperation, communication and coordination between and within organizations*
- *Quality assurance, monitoring and control systems.*

Barriers to implementation: on the basis of the above analysis, the main barriers to the implementation of the health care program should be described [7].

SOURCES OF EVIDENCE: reviews (systematic reviews and meta-analyses, narrative reviews); HTA reports; guidelines; observational studies (cohort studies, case-control studies, cross sectional studies, descriptive studies); pilot studies; best practices; own studies (e.g. questionnaires and interviews of different actors); national and international reports, registers and statistics; administrative databases (e.g. DRG); regulatory acts or programming documents (national, regional or local); reports and documents of hospital and hospital districts; clinical laboratory or manufacturer data; consensus conference; expert opinion; patients organizations documents [7].

2.3 ECONOMIC EVALUATION

The economic dimension assesses the quantity and quality of cost-effectiveness and cost-utility evidence for alternative genetic testing programs [28]. To support decision makers in fact, attention should be payed not only to the results of the economic evaluations but also to their methodology. In this way decision makers

can understand the validity of the analysis performed and its relevance to their own setting [7]. When summarizing the results of existing economic evaluations, the following information should be reported:

- Type of economic evaluation (e.g. cost effectiveness analysis, cost utility analysis, etc.)
- Testing strategy and comparator (target population, purpose, genetic test, intervention strategies)
- Perspective of the analysis (e.g. healthcare system, societal, etc.)
- Time horizon
- Measured costs
- Measured outcomes
- Discounting
- Methodological approach (i.e. using clinical studies or decision analytic modelling)
- Cost-effectiveness ratios
- Sensitivity analysis.

SOURCES OF EVIDENCE: overviews of systematic reviews of economic evaluations; systematic reviews of economic evaluations; full economic evaluations (cost-effectiveness analysis, cost utility analysis, cost-benefit analysis, cost-minimization analysis).

2.4 ETHICAL, LEGAL AND SOCIAL IMPLICATIONS

The ELSI evaluation component is concerned with the moral value that society confers on the proposed interventions, the specific related legal norms and the impact on the social life of the patient and his or her family[9]. In this section a brief overview of the major ELSI related to the genetic test under assessment should be provided. Some examples are provided below:

- *Autonomy*
  Autonomy refers to the right of persons to make an informed, independent judgment about whether they wish to be tested and whether they wish to know the results of the test; moreover, it is the right of the individual to control his or her destiny and the future use of the genetic material [29] .
- *Potential of discrimination based on genetic information*
  Since genetic test results are usually included in medical records, they may be accessible to others and affect a person's insurance coverage or employment [30]. This issue is related to the issues of privacy and confidentiality.
- *Privacy and Confidentiality*
  Genetic information has enormous implications to an individual and his or her family therefore the privacy of that information is a major concern [30]. Confidentiality implies that the information provided within the relationship is given in confidence, with the expectation that it will not be disclosed to others or will be disclosed to others only within limits [29].
- *Equity*
  Equity raises questions about access to genetic tests and treatment/ prevention strategies as well as about resources allocation. A decent minimum of health care for all should be guaranteed and

decisions about how much should be used for particular illnesses and treatments should be made [29].

- *Informed consent*
  Informed consent is an important part of the medical decision-making process as it helps ensure that patients understand the risks and benefits of health care choices [30].
- *Societal values*
  It should be considered that genetic information can raise questions about different topics such as personal responsibility, personal choice versus genetic determinism/fate, concepts of health and disease. Moreover, responses to these issues will be influenced by personal factors, family values, and community and cultural beliefs [30]. The issue of societal values is related to the issue of psychosocial impact.
- *Psychosocial impact*
  Every individual will respond differently to news of his or her genetic test results whether negative or positive and this response may involve several levels such as the individual level, family level, or community and society level. Adequate support, such as referrals to genetic counselors, psychologists, or social workers should be made as needed [30].
- *Reproductive issues*
  Genetic information is routinely used to inform reproductive decisions and medical care [30].

SOURCES OF EVIDENCE: reviews (systematic reviews and meta-analyses, narrative reviews); HTA reports; observational studies (cohort studies, case-control studies, cross sectional studies, descriptive studies); pilot studies; surveys; qualitative research; law, rules and regulations; consensus conference; expert opinion, patients organizations documents.

2.5 PATIENT PERSPECTIVE

The patient perspective dimension assesses the perspective of patients that, being the direct beneficiaries of genetic programs, can contributes to define their value [31–33].

Our framework requires to derive evidence on patient perspective from both quantitative and qualitative studies, referring to surveys or interviews incorporating the so called "patient reported outcomes". This is the only way to learn about experiences, attitudes beliefs and expectations of living with an illness and using a genetic service.

SOURCES OF EVIDENCE: reviews (systematic reviews and meta-analyses, narrative reviews); HTA reports observational studies (cohort studies, case-control studies, cross sectional studies, descriptive studies); pilot studies; surveys, qualitative research; patients' organizations documents.

SECTION 3– RESEARCH PRIORITIES

This section deals with an important challenge: the lack of scientific evidence on which to base the evaluation of genetic test. In this section evidence gaps should be identified and formulated in research questions for future research. The following table can be used as a guide (Table A).

Table A Evidence gaps and research priorities

Evaluation dimension	Evidence amount [1]	Research questions
Analytic validity		
Clinical validity		
Clinical utility		
Personal utility		
Organizational aspects		
Economic evaluation		
ELSI		
Patient perspective		

[1] Evidence amount can be defined as exhaustive/incomplete/missing

SECTION 4 – DECISION POINTS

Section IV suggests three criteria for summarizing the evidence collected and supporting the decision-making process regarding the use of the test: the net benefit, cost-effectiveness and feasibility of the delivery option under study. Moreover, the quality of the supporting evidence, measured trough validated instruments, should be taken into account.

4.1 NET BENEFIT

The net benefit of an intervention is the balance between its benefits and harms. To justify an intervention, benefits should adequately exceed harms.

The evaluative dimensions to be considered in order to assess the net benefit of a genetic testing program are: analytic validity, clinical validity, clinical utility, personal utility, ELSI, and patient perspective.

Based on these dimensions, the net benefit can be assigned to one of four classes (A–D), ranging in descending order from positive to negative (Table B). Each class should be weighted for the quality of the evidence. Specifically, the quality of the evidence should be assessed using validated instruments and a minus or plus sign (indicating low or high quality, respectively) should be added to each rating class, as appropriate.

Table B Rating of the net benefit

Rating	Meaning	Explanation
A	High chance that the implementation of the healthcare program will have a positive net benefit	Scientific evidence in favor of *analytic validity*, *clinical validity* and *clinical utility* and overall positive regarding *personal utility*, *ELSI* and *patient perspective*
B	Moderate chance that the implementation of the healthcare program will have a positive net benefit	Scientific evidence in favor of *analytic validity*, *clinical validity* and *clinical utility* but uncertain regarding personal utility, ELSI and patient perspective.
C	High/moderate chance that the implementation of the healthcare program will have a weak positive/ null net benefit	Scientific evidence in favor of *analytic validity* and *clinical validity* but uncertain for/ against clinical utility
D	High/moderate chance that the implementation of the healthcare program will have a negative net benefit	Scientific evidence uncertain for/ against clinical validity and against clinical utility

4.2 COST-EFFECTIVENESS

Economic evaluation should help decision makers answering the question "Are we satisfied that the additional healthcare resources, required to make the genetic test available to those who could benefit from it, should be spent in this way rather than some other ways?"[34].

The answer to this question depends critically on the health expected to be given up as a consequence of the incremental cost i.e. the opportunity cost. The assessment of this type of opportunity cost is commonly described as a cost-effectiveness threshold which can be compared to the incremental cost-effectiveness ratio (ICER) of the genetic test under assessment [34]. An ICER is calculated by dividing the difference in total costs (incremental cost) by the difference in the chosen measure of health outcome or effect (incremental effect) for the two alternatives, to provide a ratio of "extra cost per extra unit of health effect" [35]. A number of possible threshold values have been suggested in different context. For example the United Kingdom's National Institute for Health and Care Excellence has used for years a cost–effectiveness threshold of between 20 000 and 30 000 pounds sterling while the USA a threshold of US$ 100 000 or US$ 150 000 [36,37].

Four classes (A-D) of cost-effectiveness can be defined, ranging in descending order from highly cost-effective to not cost-effective (Table C). Each class should be weighted for the quality of the evidence. Specifically, the quality of the evidence should be assessed using validated instruments and a minus or plus sign (indicating low or high quality, respectively) should be added to each rating class, as appropriate.

Table C Rating of the cost-effectiveness

Rating	Meaning	Explanation
A	Genetic healthcare program highly cost-effective	Less costly and more effective than the alternative (cost-saving)
B	Genetic healthcare program cost-effective	More costly and more effective than the alternative (ICER less than the reference threshold)
C	Genetic healthcare program arguably cost-effective	More costly and more effective than the alternative (ICER more than the reference threshold)
D	Genetic healthcare program not cost-effective	More costly and less effective than the alternative (dominated)

4.3 FEASIBILITY

The feasibility of a genetic testing programs is determined from the organizational analysis. It can be defined as the probability of overcoming the identified barriers to implementation and can be rated in three classes (I–III) in descending order from easy to impossible (Table D).

Table D Rating of the feasibility

Rating	Meaning	Explanation
I	Feasible provision	Barriers to implementation can be easily overcome
II	Potentially feasible provision	Barriers to implementation can be overcome
III	Unfeasible provision	Barriers to implementation cannot be overcome
NA	Insufficient information	Additional information are needed

REFERENCES

[1] Burke W, Atkins D, Gwinn M, et al. Genetic test evaluation: information needs of clinicians, policy makers, and the public. Am J Epidemiol. 2002;156:311–8.

[2] Haddow J, Palomaki G. ACCE: A Model Process for Evaluating Data on Emerging Genetic Tests. In: Khoury M, Little J, Burke W, editors. Human Genome Epidemiology: A Scientific Foundation for Using Genetic Information to Improve Health and Prevent Disease. Oxford: Oxford University Press; 2004. pp 217–233 .

[3] Burke W, Zimmern R. Moving beyond acce: An Expanded Framework for Genetic Test Evaluation. 2007. Available from: http://www.phgfoundation.org/documents/369_1409657043.pdf Cited 6 June 2018.

[4] Gene Dossiers - UK Genetic Testing Network. Available from: https://ukgtn.nhs.uk/find-a-test/gene-dossiers Cited 6 June 2018.

[5] National Academies of Sciences, Engineering and Medicine. An Evidence Framework for Genetic Testing. Washington, D.C.: National Academies Press; 2017.

[6] Burke W, Zimmern RL, Kroese M. Defining purpose: a key step in genetic test evaluation. Genet Med. 2007;9:675–81.

[7] EUnetHTA Joint Action 2 WP 8. HTA Core Model ® version 3.0 2013. Available from: http://eunethta.eu/sites/5026.fedimbo.belgium.be/files/HTACoreModel3.0.pdf Cited 6 June 2018.

[8] Burke W. Genetic tests: clinical validity and clinical utility. Curr Protoc Hum Genet, 2014;81:9.15.1-8.

[9] Zimmern RL, Kroese M. The evaluation of genetic tests. J Public Health (Bangkok). 2007;29:246–50.

[10] Sun F, Bruening W, Erinoff E, Schoelles KM. Addressing Challenges in Genetic Test Evaluation. Rockville: Agency for Healthcare Research and Quality (US); 2011.

[11] Becker F, van El CG, Ibarreta D, et al. Genetic testing and common disorders in a public health framework: how to assess relevance and possibilities. Eur J Hum Genet 2011;19(Suppl 1):S6-44.

[12] Ferreira-Gonzalez A, Teutsch S, Williams MS, et al. US system of oversight for genetic testing: a report from the Secretary's Advisory Committee on Genetics, Health and Society. Per Med. 2008;5(5):521–8.

[13] Teutsch SM, Bradley LA, Palomaki GE, et al. The Evaluation of Genomic Applications in Practice and Prevention (EGAPP) Initiative: methods of the EGAPP Working Group. Genet Med. 2009;11:3–14.

[14] Zimmern RL. Testing challenges: evaluation of novel diagnostics and molecular biomarkers. Clin Med. 2009;9:68–73.

[15] Khoury MJ, McBride CM, Schully SD, et al. The Scientific Foundation for personal genomics: recommendations from a National Institutes of Health-Centers for Disease Control and Prevention multidisciplinary workshop. Genet Med. 2009;11:559–67.

[16] Grosse SD, Khoury MJ. What is the clinical utility of genetic testing? Genet Med. 2006;8:448–50.

[17] Bunnik EM, Janssens ACJW, Schermer MHN. Personal utility in genomic testing: is there such a thing? J Med Ethics. 2015;41:322–6.

[18] Kohler JN, Turbitt E, Biesecker BB. Personal utility in genomic testing: a systematic literature review. Eur J Hum Genet. 2017;25:662–8.

[19] Kohler JN, Turbitt E, Lewis KL, et al. Defining personal utility in genomics: A Delphi study. Clin Genet 2017;92:290–7.

[20] Unim B, Lagerberg T, Pitini E, et al. Identification of Delivery Models for the Provision of Predictive Genetic Testing in Europe: Protocol for a Multicentre Qualitative Study and a Systematic Review of the Literature. Front Public Heal. 2017;5:223.

[21] D'Andrea E, Marzuillo C, De Vito C, et al. Which BRCA genetic testing programs are ready for implementation in health care? A systematic review of economic evaluations. Genet Med. 2016;18:1171–80.

[22] Di Marco M, D'Andrea E, Panic N, et al. Which Lynch syndrome screening programs could be implemented in the "real world"? A systematic review of economic evaluations [e-pub ahead of print January 4, 2018]. Genet Med. 2018. doi:10.1038/gim.2017.244.

[23] Battista RN, Blancquaert I, Laberge A, van Schendel N, Leduc N. Genetics in Health Care: An Overview of Current and Emerging Models. Public Health Genomics. 2012;15:34–45.

[24] Gu Y, Warren JR, Day KJ. Unleashing the power of human genetic variation knowledge: New Zealand stakeholder perspectives. Genet Med. 2011;13:26–38.

[25] Rousseau F, Lindsay C, Charland M, et al. Development and description of GETT: a Genetic testing Evidence Tracking Tool. Clin Chem Lab Med. 2010;48:1397–407.

[26] Giacomini M, Miller F, Browman G. Confronting the "Gray Zones" of Technology Assessment: evaluating genetic testing services for public insurance coverage in Canada. Int J Technol Assess Health Care. 2003;19:301–16.

[27] Gutiérrez-Ibarluzea I. Personalised Health Care, the need for reassessment. A HTA perspective far beyond cost-effectiveness. Ital J Public Health. 2012;9.

[28] Hornberger J, Doberne J, Chien R. Laboratory-Developed Test—SynFRAME: An Approach for Assessing Laboratory-Developed Tests Synthesized from Prior Appraisal Frameworks. Genet Test Mol Biomarkers. 2012;16:605–14.

[29] Andrews L, Fullarton J, Holtzman N. Social, Legal, and Ethical Implications of Genetic Testing. Assessing Genetic Risks: Implications for Health and Social Policy, Washington, D.C.: National Academies Press; 1994.

[30] The New York-Mid-Atlantic Consortium for Genetic and Newborn Screening Services. Ethical, Legal, and Social Issues. Washington, DC: Genetic Alliance; 2010.

[31] Gagnon M, Desmartis M, Lepage-Savary D, et al. Introducing patients' and the public's perspectives to health technology assessment: A systematic review of international experiences. Int J Technol Assess Health Care. 2011;27:31–42.

[32] Facey K, Boivin A, Gracia J, et al. Patients' perspectives in health technology assessment: A route to robust evidence and fair deliberation. Int J Technol Assess Health Care. 2010;26:334–40.

[33] D'Andrea E, Lagerberg T, De Vito C, et al. Patient experience and utility of genetic information. A cross-sectional study among patients tested for cancer susceptibility and thrombophilia. Eur J Hum Genet. 2018;26:518–26.

[34] Drummond M, Sculpher M, Claxton K, Torrance G, Stoddart G. Methods for the economic evaluation of health care programmes (4th ed). New York: Oxford University Press; 2015.

[35] York Health Economics Consortium. Incremental Cost-Effectiveness Ratio (ICER) - York Health Economics Consortium 2016. Available from: http://www.yhec.co.uk/glossary/incremental-cost-effectiveness-ratio-icer Cited 6 June 2018.

[36] Bertram MY, Lauer JA, De Joncheere K, et al. Cost-effectiveness thresholds: pros and cons. Bull World Health Organ. 2016;94:925–30.

[37] Marseille E, Larson B, Kazi DS, Kahn JG, Rosen S. Thresholds for the cost–effectiveness of interventions: alternative approaches. Bull World Health Organ. 2015;93(2):118–24.
