# Supplementary material for: A proposal of a new evaluation framework towards implementation of genetic tests
Source: PLoS One. 2019 Aug 5;14(8):e0219755. doi: 10.1371/journal.pone.0219755 (PMC6681956; doi:10.1371/journal.pone.0219755)
Supplement: S2 File — (PDF) [file pone.0219755.s002.pdf]

SUPPORTING INFORMATION 2

DELPHI SURVEY QUESTIONNAIRE

Table of contents

Delphi survey questionnaire Italian (original version) p.2

Delphi survey questionnaire English translation p.5

DELPHI SURVEY QUESTIONNAIRE ITALIAN (ORIGINAL VERSION)

Delphi survey round I

QUESTIONARIO PARTE 1. MODELLI DI VALUTAZIONE PER I TEST GENETICI

Se è a conoscenza di modelli di valutazione per i test genetici, la preghiamo di indicarli nello spazio sottostante:<br>-<br>-<br>-<br>Se esiste un sito web relativo ai modelli da Lei indicati, lo riporti con l'URL nello spazio sovrastante. Se è in possesso delle pubblicazioni relative ai modelli da Lei indicati, può allegarle cliccando su allega.

QUESTIONARIO PARTE 2. DIMENSIONI VALUTATIVE

Valuti l'importanza delle seguenti componenti di valutazione dei test genetici attribuendo a ciascuna di esse un punteggio da 1 a 5 (1= minima importanza; 5= massima importanza)

DIMENSIONI VALUTATIVE	PUNTEGGIO	NOTE
1. **Inquadramento del test e della condizione clinica in esame:** il primo passo per valutare un test genetico è quello di definirlo e contestualizzarlo, riportando informazioni precise riguardo:<br>• il genotipo che deve essere studiato (ereditarietà, prevalenza, penetranza, etc.);<br>• la patologia in questione (storia naturale, caratteristiche cliniche, epidemiologia, genetica, gestione clinica, etc.);<br>• il farmaco in questione se si tratta di un test farmacogenetico (farmacodinamica, farmacocinetica, modalità di somministrazione, effetti avversi, etc.);<br>• le caratteristiche tecniche del test (materiali, conformità alle norme, etc.);<br>• l'obiettivo del test e gli interventi disponibili in base al risultato (es. ridurre la mortalità in un paziente che in base al risultato del test può beneficiare di un trattamento precoce o fornire informazioni che possono guidare le scelte riproduttive);<br>• la popolazione target cui viene sottoposto il test (età, sesso, etnia, prevalenza della patologia, etc.).	☐1 ☐2 ☐3 ☐4 ☐5	

2. **Validità analitica:** valuta la capacità del test di determinare con accuratezza, precisione ed affidabilità la mutazione genetica o i marcatori genomici di interesse. Si riferisce alla performance del test in laboratorio e comprende due elementi fondamentali:<br>• sensibilità analitica (probabilità che il test sia positivo in presenza di mutazione);<br>• specificità analitica (probabilità che il test sia negativo in assenza di mutazione).	1 2 3 4 5
3. **Validità clinica:** valuta la capacità del test di predire con accuratezza la presenza o l'assenza di un determinato fenotipo (quadro patologico). Richiede robusti studi epidemiologici e comprende tre elementi fondamentali:<br>• sensibilità clinica (probabilità che il test sia positivo in individui con la patologia in esame);<br>• specificità clinica (probabilità che il test sia negativo in individui senza la patologia in esame);<br>• valore predittivo positivo e negativo (probabilità che un individuo risultato positivo al test abbia effettivamente la patologia in esame o che un individuo risultato negativo al test non abbia effettivamente la patologia).	1 2 3 4 5
4. **Utilità clinica:** valuta l'impatto dell'introduzione del test nella pratica clinica sulla salute del paziente, in termini di miglioramento dei diversi *outcome* (sopravvivenza, morbilità, qualità della vita, soddisfazione, etc.). La valutazione considera la disponibilità, l'efficacia e i rischi degli interventi da attuare in base al risultato del test rispetto alla pratica corrente.	1 2 3 4 5
5. **Modelli di offerta:** valuta il contesto generale in cui il test genetico è offerto alle persone e alle famiglie con o a rischio di malattia genetica, prendendone in considerazione gli aspetti fondamentali:<br>• il programma di cura, definito da tutti gli interventi che precedono e seguono il test genetico;<br>• il percorso clinico del paziente tra i vari professionisti sanitari;<br>• il livello di cura in cui tali programmi e percorsi sono integrati (e.g., assistenza primaria, assistenza specialistica).	1 2 3 4 5
6. **Aspetti organizzativi:** valuta quali risorse (attrezzature, materiali, infrastrutture, personale, denaro, capacità, formazione, etc.) sono necessarie all'attuazione del test genetico e dei conseguenti interventi nella popolazione di riferimento, quali di queste sono disponibili, quali da acquisire, come mobilizzarle ed organizzarle (programmazione e gestione) e quali sono la sostenibilità economico-finanziaria e le conseguenze per il sistema sanitario nel suo complesso.	1 2 3 4 5

7. **Valutazione economica:** valuta la presenza e la qualità delle evidenze di costo-efficacia, costo-utilità etc. per il test genetico in esame.	1 2 3 4 5
8. **Implicazioni etiche, legali e sociali: c**onsiderano il valore morale che la società conferisce agli interventi proposti, le specifiche norme giuridiche correlate e l'impatto sulla vita sociale del paziente e della sua famiglia	1 2 3 4 5

9. Se ritiene necessario considerare ulteriori componenti di valutazione, oltre alle 9 precedentemente elencate, La preghiamo di indicarle nello spazio sottostante:

-
-
-

Delphi survey round II

DIMENSIONI VALUTATIVE

Valuti l'importanza della seguente componenti di valutazione dei test genetici attribuendole (1= minima importanza; 5= massima importanza)

1. **Punto di vista dei cittadini/pazienti: valuta** la prospettiva dei cittadini, dei pazienti e dei *caregivers* rispetto al test genetico e l'impatto di quest'ultimo sulla loro vita quotidiana.	1 2 3 4 5

DELPHI SURVEY QUESTIONNAIRE ENGLISH TRANSLATION

Delphi survey round I

QUESTIONNAIRE PART 1. GENETIC TESTS EVALUATION FRAMEWORK

Please list below the existing frameworks for the evaluation of genetic tests you are aware of:<br>-<br>-<br>-<br>Please, also report the URL of related websites or upload related publications if available.

QUESTIONNAIRE PART 2. EVALUATION DIMENSIONS

Please, rate on a five-point scale ranging from 1 (minimal importance) to 5 (utmost importance), the following genetic tests evaluation dimensions:

EVALUATION DIMENSIONS	SCORE	NOTES
1. **Test and clinical condition overview:** aims at introducing the clinical condition and the genetic test under examination with a brief description of:<br>• The genotype under study (heredity, prevalence, penetrance, etc.);<br>• The condition under study (natural history, clinical features, epidemiology, genetics, clinical management, etc.);<br>• The drug under study, in the case of a pharmacogenetic test (pharmacodynamics, pharmacokinetics, mode of administration, adverse effects, etc.);<br>• The genetic test technical features (materials, compliance with standards, etc.);<br>• The purpose of the test and the available interventions based on test results (e.g. reduce mortality in a patient who, based on the test result, can benefit from early treatment or provide information that can guide reproductive choices);<br>• The genetic test target population (age, sex, ethnicity, prevalence of the disease, etc.).	1 2 3 4 5	
2. **Analytic validity:** refers to the accuracy with which a particular genetic characteristic, such as a DNA sequence variant,	1 2 3 4 5	

	chromosomal deletion, or biochemical indicator, is identified in a given laboratory test. It assesses test performance in the laboratory as opposed to the clinic and includes two main elements:<br>• Analytic sensitivity (how effectively a test can detect all true positive specimens, as determined by a reference method);<br>• Analytic specificity (the ability of a measurement procedure to measure solely the analyte of interest)	
3.	**Clinical validity:** assesses the ability of the test to accurately and reliably detect or predict a clinical condition. It includes tree main elements:<br>• Clinical sensitivity (the proportion of individuals for whom the test result correctly identifies or predicts the presence of a well-defined disorder);<br>• Clinical specificity (the proportion of individuals for whom the test result correctly detects or predicts the absence of a well-defined clinical disorder);<br>• Positive and negative clinical predictive value: the probabilities that people (within a defined population) with positive test results will get the disease (positive predictive value) and that people (within a defined population) with negative results will not get the disease (negative predictive value).	1 2 3 4 5
4.	**Clinical utility:** refers to the health impact of a genetic test in terms of risk and benefit, compared to the current practice. Specifically, it measures the improvement in health outcomes - such as mortality, morbidity, or disability – due to the interventions adopted on the base of test results and the risks that might occur	1 2 3 4 5
5.	**Delivery models:** the broad context in which genetic tests are offered to individuals and families with or at risk of genetic disorders. It includes tree main aspects:<br>• The healthcare program, i.e. any activity preceding or following the genetic test;<br>• The patients' pathway through different professionals;<br>• the level of care in which the provision of the genetic healthcare program is integrated and coordinated (e.g., primary care, specialist care).	1 2 3 4 5
7.	**Organizational aspects:** estimates the expected demand for the genetic test under study and the resources needed to implement the related healthcare program; it should also consider possible barriers to implementation and further requirements.	1 2 3 4 5

9. **Economic evaluation:** assesses the quantity and quality of cost-effectiveness and cost-utility evidence for alternative genetic testing programs	1 2 3 4 5
10. **Ethical, legal and social implications:** consider the moral value that society confers on the proposed interventions, the specific related legal norms and the impact on the social life of the patient and his or her family	1 2 3 4 5

9. If you consider it necessary to consider further evaluation components, in addition to the 9 previously listed, please indicate them in the space below:

-
-
-

Delphi survey round II

EVALUATION DIMENSIONS

Please, rate on a five-point scale ranging from 1 (minimal importance) to 5 (utmost importance), the following genetic tests evaluation dimension:

2. **Citizens/Patients point of view:** assesses the perspective of citizens, patients and caregivers with respect to genetic testing and its impact on their daily lives.	1 2 3 4 5
